# Elimination of the Vesicular Acetylcholine Transporter in the Striatum Reveals Regulation of Behaviour by Cholinergic-Glutamatergic Co-Transmission

**DOI:** 10.1371/journal.pbio.1001194

**Published:** 2011-11-08

**Authors:** Monica S. Guzman, Xavier De Jaeger, Sanda Raulic, Ivana A. Souza, Alex X. Li, Susanne Schmid, Ravi S. Menon, Raul R. Gainetdinov, Marc G. Caron, Robert Bartha, Vania F. Prado, Marco A. M. Prado

**Affiliations:** 1 Molecular Brain Research Group, Robarts Research Institute, Schulich School of Medicine & Dentistry, University of Western Ontario, London, Ontario, Canada; 2 Department of Physiology and Pharmacology, Schulich School of Medicine & Dentistry, University of Western Ontario, London, Ontario, Canada; 3 Department of Anatomy & Cell Biology, Schulich School of Medicine & Dentistry, University of Western Ontario, London, Ontario, Canada; 4 Program in Molecular Pharmacology, Faculty of Medicine, Federal University of Minas Gerais, Belo Horizonte, Minas Gerais, Brazil; 5 Imaging Research Group, Robarts Research Institute, Schulich School of Medicine & Dentistry, University of Western Ontario, London, Ontario, Canada; 6 Department of Medical Biophysics, Schulich School of Medicine & Dentistry, University of Western Ontario, London, Ontario, Canada; 7 Department of Neuroscience and Brain Technologies, Italian Institute of Technology, Genova, Italy; 8 Department of Cell Biology, Duke University Medical Center, Durham, North Carolina, United States of America; Mount Sinai School of Medicine, United States of America

## Abstract

Cholinergic neurons in the striatum are thought to play major regulatory functions in motor behaviour and reward. These neurons express two vesicular transporters that can load either acetylcholine or glutamate into synaptic vesicles. Consequently cholinergic neurons can release both neurotransmitters, making it difficult to discern their individual contributions for the regulation of striatal functions. Here we have dissected the specific roles of acetylcholine release for striatal-dependent behaviour in mice by selective elimination of the vesicular acetylcholine transporter (VAChT) from striatal cholinergic neurons. Analysis of several behavioural parameters indicates that elimination of VAChT had only marginal consequences in striatum-related tasks and did not affect spontaneous locomotion, cocaine-induced hyperactivity, or its reward properties. However, dopaminergic sensitivity of medium spiny neurons (MSN) and the behavioural outputs in response to direct dopaminergic agonists were enhanced, likely due to increased expression/function of dopamine receptors in the striatum. These observations indicate that previous functions attributed to striatal cholinergic neurons in spontaneous locomotor activity and in the rewarding responses to cocaine are mediated by glutamate and not by acetylcholine release. Our experiments demonstrate how one population of neurons can use two distinct neurotransmitters to differentially regulate a given circuitry. The data also raise the possibility of using VAChT as a target to boost dopaminergic function and decrease high striatal cholinergic activity, common neurochemical alterations in individuals affected with Parkinson's disease.

## Abstract 2

A novel mouse model that eliminates cholinergic neurotransmission in the striatum while leaving glutamate release intact reveals differential effects on cocaine-induced behavior and dopaminergic responses.

## Author Summary

The neurotransmitters dopamine and acetylcholine play opposite roles in the striatum (a brain region involved in motor control and reward-related behaviour), and their balance is thought to be critical for striatal function. Acetylcholine in the striatum has been linked to a number of functions, including control of locomotor activity and response to drugs of abuse. However, striatal cholinergic interneurons can also release glutamate (in addition to acetylcholine) and it is presently unclear how these two neurotransmitters regulate striatal-dependent behaviour. Previous work has attempted to resolve this issue by ablating cholinergic neurons in the striatum, but this causes loss of both cholinergic and glutamatergic neurotransmission. In this study, we created a novel genetic mouse model which allowed us to selectively interfere with secretion of acetylcholine in the striatum, while leaving total striatal glutamate release intact. In these mice, we observed minimally altered behavioural responses to cocaine, suggesting that striatal glutamate, rather than acetylcholine, is critical for cocaine-induced behavioural manifestations. Furthermore, elimination of striatal acetylcholine release affects how striatal output neurons respond to dopamine, by up-regulating dopaminergic receptors and changing behavioural responses to dopaminergic agonists. Our experiments highlight a previously unappreciated physiological role of cholinergic-glutamatergic co-transmission and demonstrate how a population of neurons can use two distinct neurotransmitters to differentially regulate behaviour.

## Introduction

The striatum is the major input gateway to the basal ganglia. Striatal activity plays important roles in controlling motor functions and goal-directed and reward-related behaviours [1]–[4]. The striatum is the brain region mostly affected in motor diseases, such as Parkinson's disease (PD), Huntington's disease, and dystonia [5]. Medium spiny GABAergic neurons (MSN), activated by corticostriatal glutamatergic inputs, are the major output neurons for the striatum; these neurons are regulated extensively by the classical neurotransmitters dopamine and acetylcholine (ACh) [1],[2],[4],[6]. These two neurotransmitters have reciprocal relationships, regulating each other's release at different levels, and they generally have opposing actions in the direct and indirect striatal pathways [1],[5],[7]–[9]. Regulation of MSNs by dopamine has received considerable attention, largely due to the well-known effects of reduced dopamine levels leading to motor symptoms in PD [10] and the role of dopamine in the effect of drugs of abuse [11].

In contrast to the widely known effects of dopamine in the striatum, we know considerably less about how ACh shapes striatal function. Cholinergic neurons form a small population of aspiny and large striatal interneurons that provide the sole source of cholinergic innervation to MSNs [12],[13]. These neurons fire constantly and therefore ensure relatively high levels of extracellular ACh. To maintain high levels of transmitter release, cholinergic neurons transport ACh synthesized in the cytoplasm into synaptic vesicles, a process which requires the activity of the vesicular acetylcholine transporter (SLC18A3, VAChT [14],[15]), the last cholinergic-specific step for ACh-mediated neurotransmission [16]. A variety of muscarinic receptors [17], as well as nicotinic subtypes of receptors [18]–[21], involved in controlling striatal function add complexity to unravelling the role of endogenous ACh in the striatum. To make matters more difficult, several central cholinergic neurons express both VAChT and distinct vesicular glutamate transporters (VGLUTs) and thus are able to store and release both ACh and glutamate [22]. Striatal cholinergic neurons express VGLUT3 [23]–[26] and simultaneously release glutamate and ACh [27]. It is unknown, however, if cholinergic neurons can use both neurotransmitters to regulate striatal function.

Elimination of cholinergic neurons in the striatum, using ablation strategies, indicated that these neurons have a role in regulating spontaneous and cocaine-induced locomotor activity, as well as its rewarding properties [28]–[31]. These neurons have the capacity to release both ACh and glutamate; therefore, non-selective manipulations of striatal cholinergic neurons can affect both VAChT and VGLUT-mediated neurotransmission. Interestingly, mice null for VGLUT3 phenocopy many of the behavioural alterations found in mice that had their accumbens cholinergic neurons ablated [25]. However, because VGLUT3-null mice also presented a 40% decrease on acetylcholine release, it is difficult to discern the individual effects of these two neurotransmitters. Therefore, the specific roles of ACh for striatal function have not yet been addressed.

To investigate the possibility that cholinergic neurons can use these two distinct neurotransmitters differentially to regulate striatal circuitry, we generated a novel mouse line in which we selectively eliminated ACh release by deleting the VAChT gene in the striatum. Our results reveal specific roles for ACh release in regulating dopamine receptor-mediated locomotor responses, but suggest that some of the previous functions attributed to these neurons are related to their ability to release glutamate.

## Results

### D2-Cre Mice Express Cre in Striatal Cholinergic Neurons

To address specific roles of ACh release in striatal function we generated a VAChT floxed mouse line (VAChT^flox/flox^, [32]), as constitutive VAChT knockout mice do not survive birth due to impaired breathing [16]. The addition of lox P sites did not change VAChT expression at the mRNA and protein levels when compared to wild-type control mice. VAChT^flox/flox^ mice had normal levels of VAChT and other pre-synaptic cholinergic markers. In addition locomotor activity, grip-strength, and fatigue were identical in VAChT^flox/flox^ mice and wild-type mice [32].

**Figure 1:**
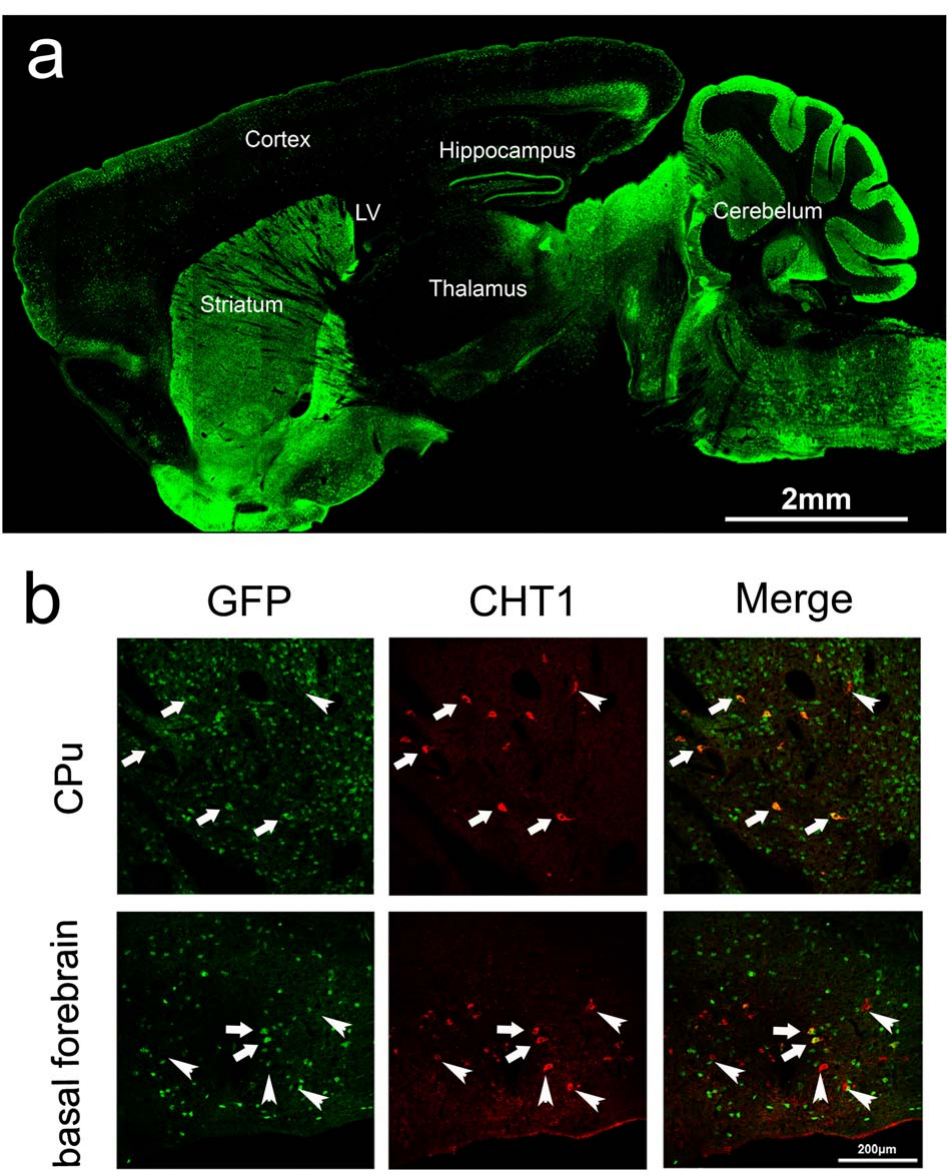
D2-Cre drives the expression of Cre in striatal cholinergic neurons. (a) Expression pattern of Cre detected by staining for YFP in the brain of D2-Cre;Rosa26-YFP mice. (b) Sections from different regions of the central nervous system were immunostained for CHT1 (Red) and YFP (Green) in D2-Cre;Rosa26-YFP mice. Arrows show localization of Cre expression (YFP) in cholinergic neurons (CHT1 staining). Arrowheads show cholinergic neurons that do not express Cre. For additional brain regions, see Figure S1 and Table S1.

In order to selectively eliminate VAChT in the striatum, we used the D2-Cre bacterial artificial chromosome (BAC) transgenic mouse line generated by GENSAT [33], which expresses the enzyme Cre recombinase under the control of regulatory elements of the D2 dopamine receptor (D2R). Details related to this mouse line, including control experiments demonstrating that the expression of Cre has no effects on the parameters studied here, are presented in Experimental Procedures and Figure S6. To test whether Cre was expressed in striatal cholinergic neurons, we crossed D2-Cre mice to *Rosa26* reporter mice (*Rosa26*-YFP mice), in which the *Rosa26* locus expresses YFP once Cre-mediated recombination has occurred (Figure 1a). We found that in D2-Cre;Rosa26-YFP mice almost 100% of striatal cholinergic neurons identified with an antibody against CHT1 also showed Cre-recombination (YFP staining 98% co-localization, Table S1). We did not detect co-localization of YFP in cholinergic neurons in the penduculopontine nucleus or in motoneurons in the brainstem (Figure S1 and Table S1). Partial localization of YFP in cholinergic neurons was detected in the basal forebrain, albeit to a much lower extent than in the striatum (approx. 50%, Figure 1b and Table S1). We therefore intercrossed D2-Cre mice to VAChT^flox/flox^ mice to generate mice with selective elimination of VAChT in the striatum (VAChT^D2-Cre-flox/flox^) or control mice (VAChT^flox/flox^). Genotyping for these lines is shown in Figure S2. VAChT^D2-Cre-flox/flox^ mice were born in the expected Mendellian ratio and did not present overt phenotypes. We found no gross morphological alterations in the striatum or other brain sections stained with hematoxylin/eosin in VAChT^D2-Cre-flox/flox^ mice compared to control mice (unpublished data).

**Figure 2:**
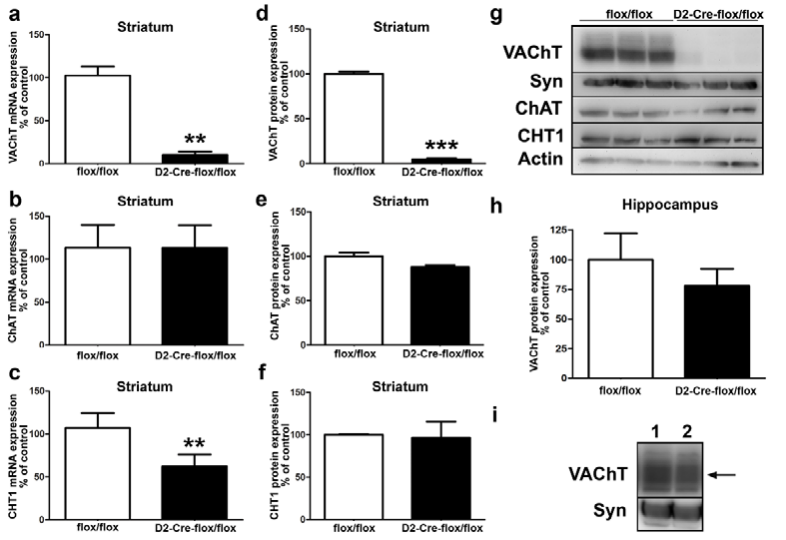
Expression of VAChT in the striatum of VAChT^D2-Cre-flox/flox^ mice. (a)VAChT mRNA expression, (b) ChAT mRNA expression, (c) CHT1 mRNA expression, (d) VAChT protein expression, (e) ChaT protein expression, (f) CHT1 protein expression, (g) representative immunoblot of control and VAChT^D2-Cre-flox/flox^ striatal tissue, (h) VAChT protein expression in the hippocampus, and (i) representative immunoblot of protein expression in the hippocampus. ** *p*<0.01 *** *p*<0.001. mRNA expression levels were quantified by qPCR using actin to normalize the data, and figures represent *N* = 5 mice. Protein levels were quantified using synaptophysin as a loading control. *N* = 5 mice. See Figure S2 for VAChT levels in the spinal cord.

**Figure 3:**
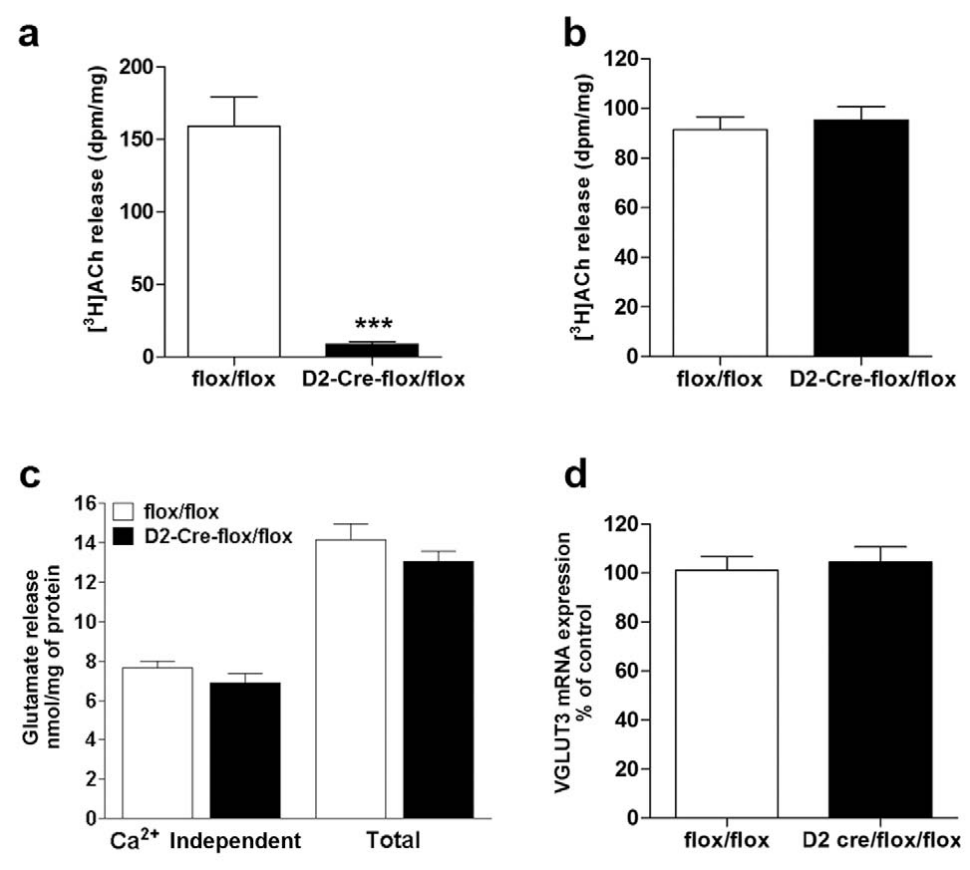
Release of acetylcholine and glutamate from VAChT^D2-Cre-flox/flox^ mice. (a) Release of [^3^H]ACh from striatal slices in response to depolarization with KCl (33 mM). Basal release was subtracted from stimulated release to obtain only evoked release. *** *p*<0.001. (b) Release of [^3^H]ACh from hippocampal slices performed as in (a). (c) Release of glutamate from striatal isolated nerve terminals and (d) expression of VGLUT 3 in the striatum. *N* = 5.

To assess the degree of Cre-mediated recombination we evaluated the expression of VAChT in the striatum of VAChT^D2-Cre-flox/flox^. As expected, based on the observations with the D2-Cre;Rosa26-YFP mice, both mRNA and protein levels for VAChT were almost abolished in the striatum of VAChT^D2-Cre-flox/flox^ (Figure 2a,d,g). In contrast, choline acetyltransferase (ChAT) and the high-affinity choline transporter (CHT1) protein levels were not altered (Figure 2e and f). There was no difference in VAChT protein expression levels in the hippocampus of VAChT^D2-Cre-flox/flox^ mice when compared to controls (Figure 2h and i). Accordingly, release of [^3^H]-ACh was abolished in striatal slices from VAChT^D2-Cre-flox/flox^ mice depolarized with high KCl, whereas it was identical to controls in hippocampal slices (Figure 3a and b).

Acetylcholine can modulate glutamate release via pre-synaptic nicotinic receptors in projection glutamatergic nerve-terminals [34]. In addition, striatal cholinergic neurons can also release glutamate [27]. Therefore, we examined if there was any effect of VAChT elimination on glutamate release. Isolated nerve terminals were obtained from striatal tissue of VAChT^D2-Cre-flox/flox^ and control mice and glutamate release was stimulated by KCl. We did not detect changes in glutamate release from isolated nerve terminals in VAChT-deficient mice compared to controls (Figure 3c). It should be noted, however, that this method does not separate terminals containing VGLUT3 from nerve terminals containing other VGLUTs, and therefore only reflects global changes in glutamate release. Moreover, VGLUT3 mRNA expression by qPCR did not differ in VAChT^D2-Cre-flox/flox^ mice compared to control mice (Figure 3d). These results suggest that overall glutamate release is not grossly altered in these mice.

### VAChT^D2-Cre-flox/flox^ Mice Do Not Show Motor Deficits

Because we detected the presence of Cre-mediated recombination in motoneurons in the spinal cord (Figure S1), which could affect the behavioural performance in VAChT^D2-Cre-flox/flox^ mice, we examined the cholinergic system in the spinal cord of VAChT^D2-Cre-flox/flox^ mice. We did not find alterations in mRNA levels for VAChT in the spinal cord of VAChT^D2-Cre-flox/flox^ mice (Figure S3). However, we detected an increase in ChAT mRNA and protein levels in the spinal cord. Surprisingly, there was also about a 50% decrease in VAChT protein levels. Previous experiments showed that up to a 50% decrease in the expression of VAChT in the spinal cord is well tolerated in mice and does not alter motor function [35],[36]. In agreement with these previous results, VAChT^D2-Cre-flox/flox^ mice showed no difference in grip-force strength (Figure S4a, *t*_(47)_ = 1.702, *p* = 0.095) or fatigue (detected by the Wire-hang task, Figure S4b, Mann-Whitney, T_(13)_ = 49, *p* = 0.710). Interestingly, we also found that relative to controls, VAChT^D2-Cre-flox/flox^ mice showed no deficit in motor performance or motor learning assessed using the rotarod test (Figure S4c, Repeated Measures ANOVA reveal no difference between the two genotypes with respect to time to fall, *F*_(1,261)_ = 0.0000409, *p* = 0.995; both sets of mice improved their performance, *F*_(9,261)_ = 41.614, *p*<0.001; and there was no interaction between genotype and session, *F*_(9,261)_ = 1.333, *p* = 0.220). These results show that despite a decrease in the levels of VAChT in the spinal cord there were no detectable changes in motor function. The rotarod experiments also suggest that VAChT^D2-Cre-flox/flox^ mice are physically fit and that motor learning does not appear to depend on striatal cholinergic activity.

### VAChT^D2-Cre-flox/flox^ Mice Do Not Show Broad Cognitive Deficits

Next, as a further control experiment, we determined if the elimination of ACh release in the striatum could interfere with cognitive performance that is believed to be generally independent of striatal function. We used object recognition memory, a task that is thought to be dependent on the hippocampus [37],[38] and perirhinal cortex [39], and has been previously shown to be sensitive to global decreases in VAChT levels [35],[36],[40]. In this test VAChT^D2-Cre-flox/flox^ mice performed identically to controls, suggesting that important cognitive functions are preserved in this new mouse line (Figure S4d, two-way ANOVA revealed no effect of genotype, *F*_(1,16)_ = 0.651, *p* = 0.431, a significant effect for object, *F*_(1,16)_ = 21.559, *p*<0.001 and no Object × Genotype interaction, *F*_(1,16)_ = 0.0185, *p* = 0.893).

Previous experiments have shown that the density of cholinergic neurons in the accumbens, as well as expression of ChAT, is decreased in the post-mortem brain of schizophrenic individuals [41],[42]. Moreover, partial ablation of cholinergic striatal neurons caused alterations in sensorimotor gating [43]. Therefore, we used habituation to acoustic startle and pre-pulse inhibition to assess sensorimotor gating, but found no effects of elimination of striatal VAChT on these parameters (Figure S5). These results show that decreased striatal ACh release does not cause sensorimotor gating dysfunctions in these animals and likely in schizophrenia as well.

### VAChT^D2-Cre-flox/flox^ Mice Have Normal Spontaneous Locomotion

**Figure 4:**
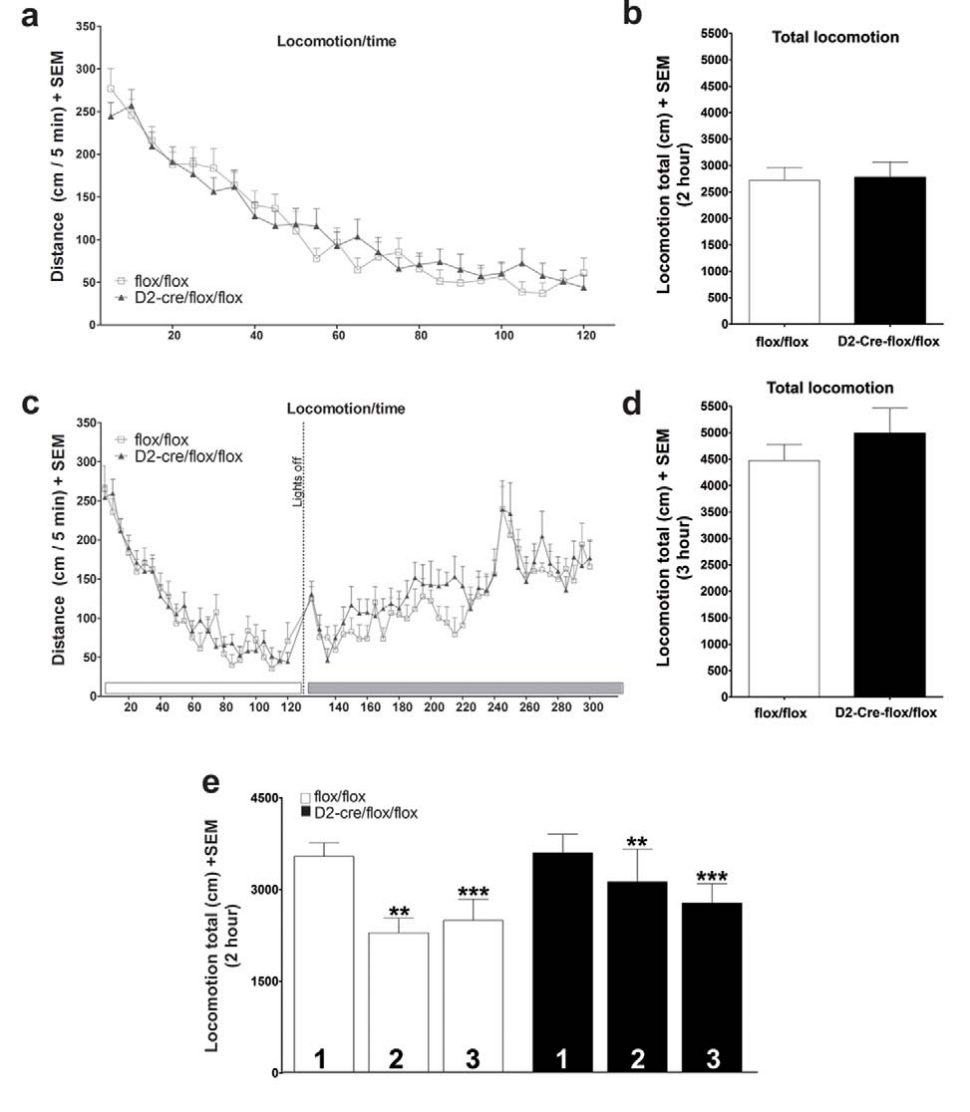
Locomotor activity of VAChT^D2-Cre-flox/flox^ mice. (a) Horizontal locomotor activity in an open-field for VAChT^D2-Cre-flox/flox^ (*N* = 24) and control mice (*N* = 27). (b) Cumulative 2 h locomotion VAChT^D2-Cre-flox/flox^ (*N* = 24) and control mice (*N* = 27) (c) dark cycle activity of VAChT^D2-Cre-flox/flox^ (*N* = 16) and control mice (*N* = 15). (d) Total locomotion activity during the first initial hours of the dark-cycle. (e) Habituation in the open-field measured as cumulative 2 h locomotion for VAChT^D2-Cre-flox/flox^ and control mice in 3 consecutive days. ** *p*<0.01, *** *p*<0.001 compared to the first day. VAChT^D2-Cre-flox/flox^ *N* = 10; control mice *N* = 21.

There are controversial views regarding the role of striatal cholinergic neurons in locomotion. Previous experiments in which cholinergic neurons in the nucleus accumbens were ablated indicated that loss of these neurons caused hyperlocomotion and increased sensitivity to the locomotor effects of cocaine [29]–[31]. However, more recent experiments using an optogenetics approach failed to detect an increased locomotor activity in mice in which striatal cholinergic neurons were acutely silenced [44]. In agreement with the latter, we found no differences in locomotor activity when we compared VAChT^D2-Cre-flox/flox^ mice to controls (Figure 4a). The dynamics of total horizontal activity (Figure 4a and 4b, *t*_(48)_ = 0.1464; *p* = 0.884) or counts of vertical activity (unpublished data, *t*_(24)_ = 1.027; *p* = 0.315) were essentially identical in the two strains. Importantly, in control experiments D2-Cre mice did not differ in locomotor activity from respective wild type mice (Figure S6).

It has been shown that VGLUT3-null mice present hyperactivity, which was attributed to decreased ACh release from striatal cholinergic neurons due to decreased filling of synaptic vesicles with ACh [25]. Because these experiments with VGLUT3-null mice were performed in the initial hours of the dark cycle, we reproduced these conditions with a new cohort of our mice. The VAChT^D2-Cre-flox/flox^ mice were no more active than their control counterparts during the first hours of the dark cycle (Figure 4c and d, repeated measures ANOVA shows no main effect of genotype, *F*_(1,1593)_ = 0.321, *p* = 0.576, significant effect of time, *F*_(59,1593)_ = 14.411, *p*<0.001 and no interaction Genotype × Time, *F*_(59,1593)_ = 0.947, *p* = 0.591; total activity was not different, Mann-Whitney, T_(29)_ = 213, *p* = 0.431). Finally, we also tested inter-session habituation by investigating locomotor activity in 3 consecutive days in the open-field (Figure 4e). We observed that both genotypes habituated similarly to the open-field. Repeated measures ANOVA confirmed that the general activity was the same for both genotypes (genotype factor, *F*_(1,58)_ = 0.932, *p* = 0.342). The activity decreased over the day (day factor, *F*_(2,58)_ = 10.244, *p*<0.001) and both genotypes habituated to the environment at comparable rates (interaction between genotype and day, *F*_(2,58)_ = 1.506, *p* = 0.230). Evidently, deletion of VAChT in the striatum does not affect general spontaneous activity or compromise the capacity to habituate to a new environment.

### Responses of VAChT^D2-Cre-flox/flox^ Mice to Cocaine

**Figure 5:**
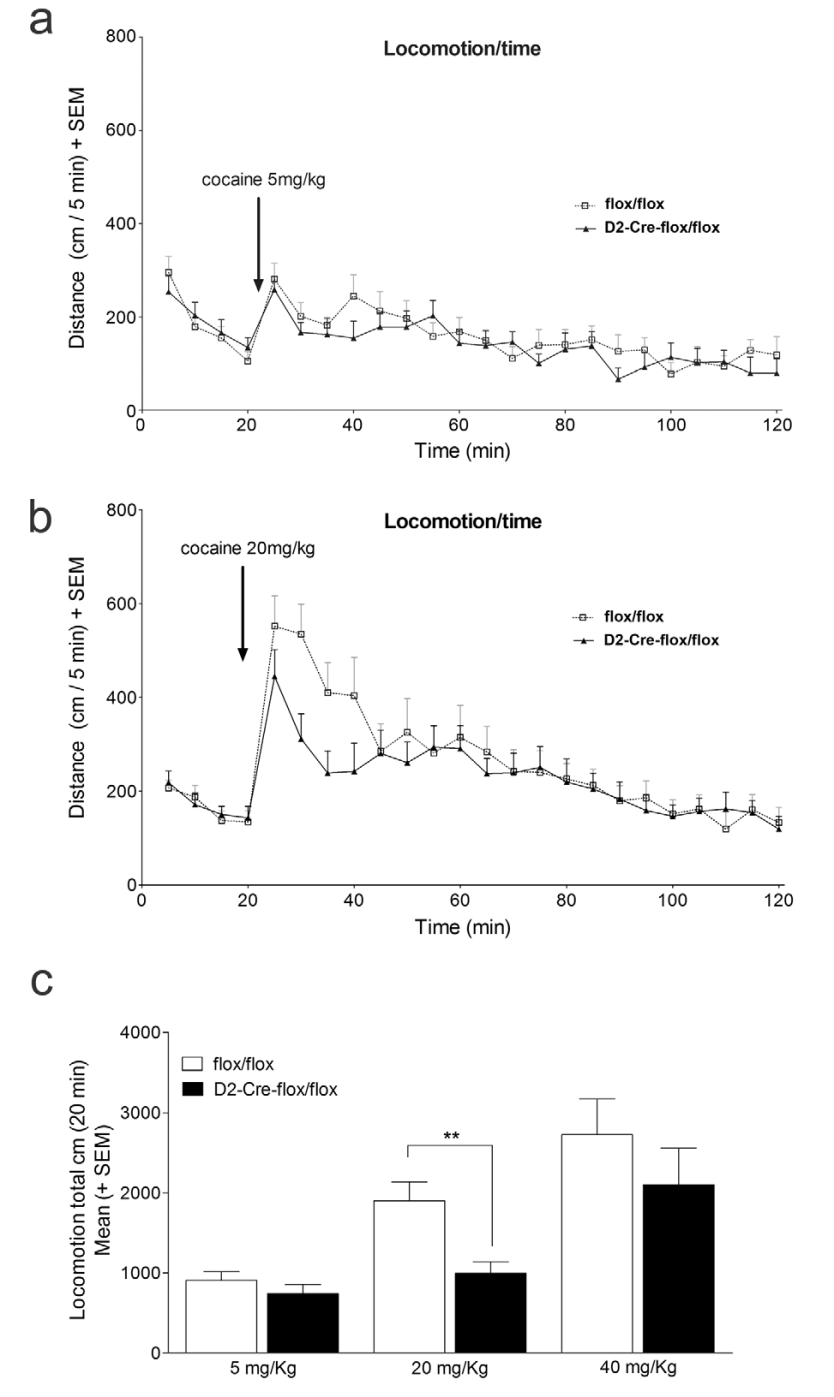
Cocaine-mediated locomotor activity in VAChT^D2-Cre-flox/flox^ mice. (a) Mice were injected with 5 mg/kg of cocaine after 20 min in the open-field and horizontal locomotor activity was measured. (b) Locomotor activity before and after injection of 20 mg/kg of cocaine. As with 5 mg/kg the mice were injected with cocaine after 20 min in the open-field. (c) Total locomotion during the 20 min following cocaine injection. ** *p*<0.01. Injection of saline did not change locomotor activity for either genotype (unpublished data). For 5 mg/kg *N* = 7 for control and 9 for VAChT^D2-Cre-flox/flox^. For 20 mg/kg *N* = 10 for control and 15 for VAChT^D2-Cre-flox/flox^ mice. For 40 mg/kg *N* = 8.

Previous experiments in mice in which cholinergic interneurons were ablated suggested that decreased ACh levels increase sensitivity of mice to the locomotor effects of cocaine [29]–[31]. However, these experiments did not separate the effects of VAChT and VGLUT3-mediated transmission. Interestingly, VGLUT3-null mice are also more sensitive to the locomotor effects of cocaine, a result that was attributed at least in part to a decrease in striatal ACh release [25]. Due to the surprising observations of normal locomotor activity in VAChT-deficient mice, we investigated the specific effects of the elimination of VAChT-mediated neurotransmission on the actions of cocaine. Administration of 5, 20, or 40 mg/kg of cocaine increased locomotor activity in VAChT^flox/flox^ mice and VAChT^D2-Cre-flox/flox^ mice (Figure 5c, two-factor ANOVAs show a significant effect of genotype, *F*_(1,51)_ = 6.531, *p* = 0.014, significant effect of treatment, *F*_(3,51)_ = 15.611, *p*<0.001, and no Genotype × Treatment interaction, *F*_(3,51)_ = 0.983, *p* = 0.381). There was no difference between the two genotypes in their ability to increase activity in response to cocaine-injected i.p. at 5 mg/kg dose (Figure 5a, 5 mg/kg, repeated measures ANOVAs show no effect of genotype, *F*_(1,322)_ = 0.201, *p* = 0.661, significant effect of time, *F*_(23,322)_ = 12.820, *p*<0.001, and no Time × Genotype interaction, *F*_(23,322)_ = 1.373, *p* = 0.121). Paradoxically, at 20 mg/kg VAChT^D2-Cre-flox/flox^ mice showed a smaller effect of cocaine in locomotor activity than controls (Figure 5b, 20 mg/kg, repeated measures ANOVA shows significant effect of genotype, *F*_(1,480)_ =  11.345, *p*<0.001, significant effect of time, *F*_(23,480)_ = 9.464, *p*<0.001, and no Time × Genotype interaction, *F*_(23,480)_ = 0.945, *p* = 0.537). Analysis of total activity counts showed a clear effect of genotype (Figure 5c, Mann-Whitney, T_(23)_ = 166.000, *p*<0.05). At 40 mg/kg both genotypes showed similar responses (Figure 5c, *t*_(14)_ = 0.980, *p* = 0.344), suggesting that lack of striatal VAChT altered the response to 20 mg/kg of cocaine, but overall did not cause increased sensitivity to locomotor effects of cocaine.

**Figure 6:**
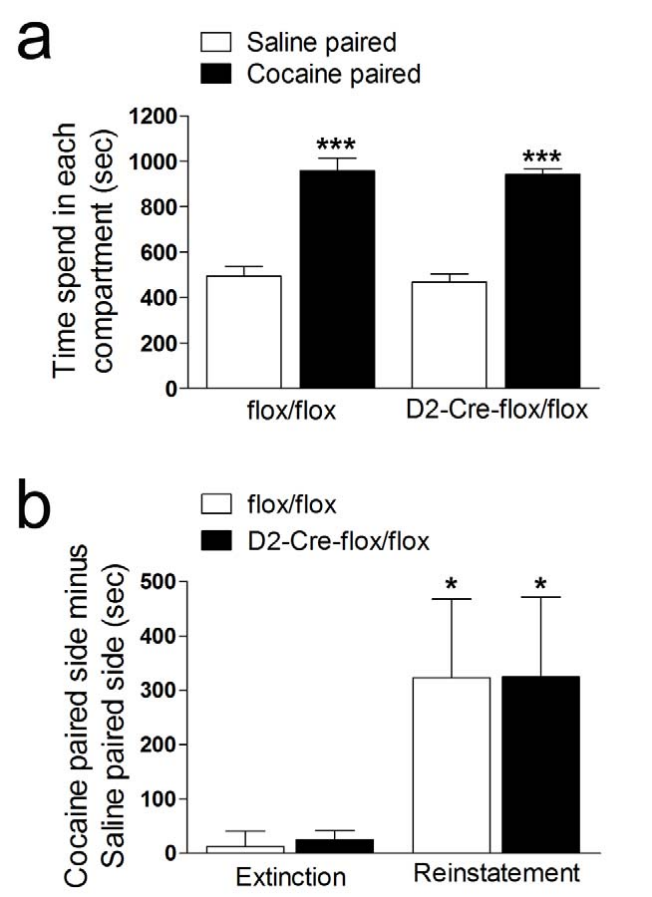
CPP response of VAChT^D2-Cre-flox/flox^ mice. (a) In the conditioning phase (days 2–7) mice received alternating injections of 20 mg/kg of cocaine or vehicle and were immediately confined into one of the two conditioning chambers for 30 min. The CPP response was measured on day 8, when the animals were allowed to move freely in the CPP apparatus and the time spent in each compartment was measured (*N* = 6). (b) Reinstatement to cocaine was tested after extinction of CPP by pairing of the cocaine paired chamber with saline injections. Once the extinction was acquired, a prime of 10 mg/kg of cocaine was injected and the animals re-exposed to the CPP apparatus. The time spent in each compartment was measured (*N* = 5). * *p*<0.05, reinstatement versus extinction. *** *p*<0.001, cocaine paired versus saline paired.

Cocaine increases firing of striatal cholinergic neurons [44] and the release of ACh in the striatum [45]–[47]. Previous experiments have suggested that striatal cholinergic neurons also play important roles in the rewarding effects of cocaine. Indeed, optogenetic silencing of striatal cholinergic neurons seemed to attenuate the response of cocaine in a conditioned-place preference (CPP) paradigm. Because these experiments did not separate the contribution of ACh from that of glutamate and to determine if there was a causal link between ACh release and expression of cocaine-induced CPP, we performed CPP experiments with VAChT^D2-Cre-flox/flox^ mice. We were unable to obtain reliable CPP with either genotype at 5 mg/kg of cocaine (unpublished data). In contrast, at 20 mg/kg we detected robust CPP in both genotypes (Figure 6a, repeated measures ANOVAs show no effect of genotype, *F*_(1,10)_ = 0.443, *p* = 0.521, significant effect of treatment, *F*_(1,10)_ = 86.033, *p*<0.001, and no Genotype × Treatment interaction, *F*_(1,10)_ = 0.0118, *p* = 0.916). In these experiments we used an extended protocol [48] with consecutive injections of cocaine in alternate days. We repeated the short protocol used before in the optogenetic experiments [44] with only one injection of cocaine (20 mg/kg), but we were unable to detect place preference in control or VAChT^D2-Cre-flox/flox^ mice (unpublished data). In addition, neither extinction of CPP nor relapse, measured as a reinstatement of CPP by a priming injection of cocaine after extinction, were altered in mice without striatal VAChT (Figure 6b, repeated measures ANOVAs show no effect of genotype, *F*_(1,7)_ = 0.00057, *p* = 0.982, significant effect of treatment, *F*_(1,7)_ = 7.457, *p* = 0.029, and no Genotype × Treatment interaction, *F*_(1,7)_ = 9.67×10^−5^, *p* = 0.992). Therefore, there was no difference in CPP response for the two genotypes.

**Figure 7:**
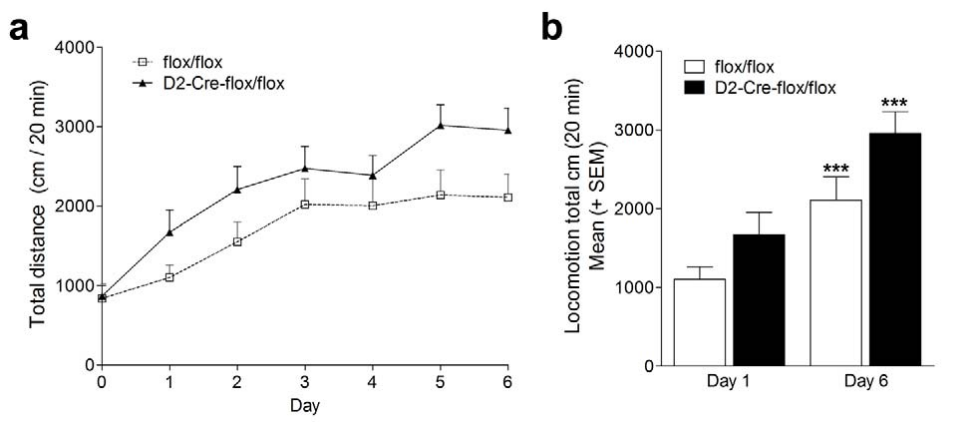
Behavioural sensitization to cocaine. (a) Repeated cocaine injections (10 mg/kg) promoted a progressive increase of locomotor sensitization (repeated measures ANOVAs show a significant effect of treatment, *F*_(1,16)_ = 33.855, *p*<0.001). VAChT^D2-Cre-flox/flox^ mice clearly manifested an enhancement in the locomotor activity in comparison with their control subjects (repeated measures ANOVAs show a significant effect of genotype, *F*_(1,16)_ = 4.902, * *p*<0.05). (b) Cumulative 20 min locomotion after cocaine injection (10 mg/kg) of VAChT^D2-Cre-flox/flox^ mice and controls (*** *p*<0.001, day 6 versus day 1). Day 0 is the basal activity of the animals (no cocaine was injected).

Behavioural sensitization protocols for cocaine likely reflect altered synaptic plasticity in response to the drug [49], which manifests as an increase in the locomotor effects of cocaine. In a separate group of mice, we measured behavioural sensitization to 10 mg/kg of cocaine (Figure 7) and found that repeated treatment with this dose of cocaine seems to cause slightly higher locomotor activity in VAChT^D2-Cre-flox/flox^ mice, but the relative increase in behavioural sensitization was not different between genotypes (Figure 7a,b, repeated measures ANOVAs show a significant effect of genotype, *F*_(1,16)_ = 4.902, *p* = 0.042, significant effect of treatment, *F*_(1,16)_ = 33.855, *p*<0.001, and no Genotype × Treatment interaction, *F*_(1,16)_ = 0.496, *p* = 0.491). Thus, elimination of striatal ACh release caused a small change in the dose-response profile of cocaine-treated mice in intermediate doses: a slight increase in activity is observed at 10 mg/kg, whereas a decrease in locomotor response is observed at 20 mg/kg in mutant mice.

### VAChT^D2-Cre-flox/flox^ Mice Have Increased Responses to Dopamine Agonists

The balance between acetylcholine-dopamine is important in a number of conditions, including PD; therefore we further investigated dopaminergic function in VAChT^D2-Cre-flox/flox^ mice. For that, we first determined the concentration of dopamine and metabolites in the striatum of VAChT^D2-Cre-flox/flox^ mice and compared these to control mice. In general there were no major changes in dopamine and metabolites in these mutant mice (Table 1). However, the ratio between dopamine and DOPAC as well as dopamine and HVA were significantly changed, showing that dopamine turnover is decreased by 25% (*p*<0.001), suggesting potential relatively minor alterations in dopamine dynamics or metabolism.

**Table 1:** Catecholamine content in the striatum (ng/100 mg of brain tissue).

	NE	DA	DOPAC	HVA	DOPAC/DA	HVA/DA	(DOPAC+HVA)/DA
fx/fx	20.400±1.515	358.600±39.300	31.480±3.523	58.140±5.205	0.088±0.001	0.164±0.004	0.250±0.006
D2-Cre-fx/fx	27.800±1.878*	365.400±27.570	25.160±1.780	48.540±3.236	0.069±0.002*	0.134±0.004*	0.200±0.004*

HPLC analysis of supernatant samples of striatum from VAChT^D2-Cre-flox/flox^ and control mice (*n* = 5). The samples were analyzed for norepinephrine (NE), dopamine (DA), and its two metabolites, 3,4-dihydroxyphenylacetic acid (DOPAC) and homovanillic acid (HVA) by NoAb BioDiscoveries. * *p*<0.05.

**Figure 8:**
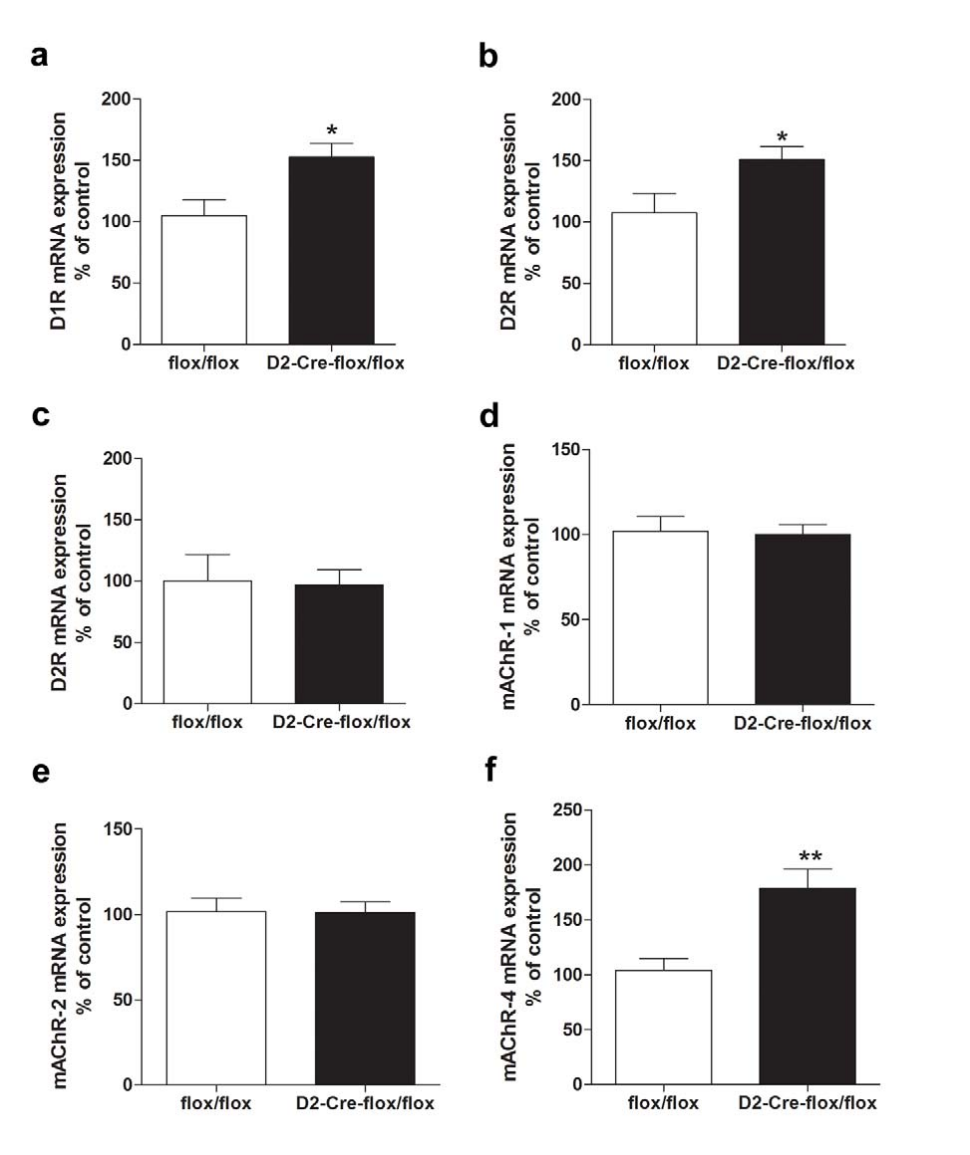
Expression of dopamine and acetylcholine muscarinic receptors in VAChT^D2-Cre-flox/flox^ mice. (a) D1R mRNA expression in striatum, (b) D2R mRNA expression in striatum, (c) D2R mRNA expression in the midbrain, (d) M1 mRNA expression in striatum, (e) M2 mRNA expression in striatum, and (f) M4 mRNA expression in striatum. * *p*<0.05 and ** *p*<0.01.

To further assess dopaminergic function, we performed qPCR analysis for D1R and D2R expression in the striatum. We detected an increase in the expression of D1R and D2R mRNAs in the striatum of VAChT^D2-Cre-flox/flox^ mice compared to control mice (Figure 8a and b, D1R, *t*_(12)_ = 2.756, *p*<0.05, D2R, *t*_(14)_ = 2.300, *p*<0.05). In contrast, D2R mRNA expression in the midbrain was not altered (Figure 8c). G-protein coupled receptors (GPCRs) can have agonist-independent effects; hence, altered expression of such receptors could modulate behaviour even in the absence of neurotransmitter release. We thus also investigated the expression of cholinergic receptors. Figure 8d–f indicates that expression of M1 and M2 muscarinc receptors (mAChR1 and mAChR2) was unchanged, whereas M4 muscarinic receptors (mAChR4) showed increased expression (*t*_(12)_ = 3.678, *p*<0.05). Homozygous mice expressing D2-BAC-GFP construct present some dopaminergic phenotypes [50], however control experiments show that the heterozygous D2-Cre mice used here do not present any of the phenotypes associated with selective elimination of striatal VAChT (Figure S6). Because dopamine receptor expression was normal in D2-Cre mice, we conclude that these molecular alterations are due to the loss of ACh release.

**Figure 9:**
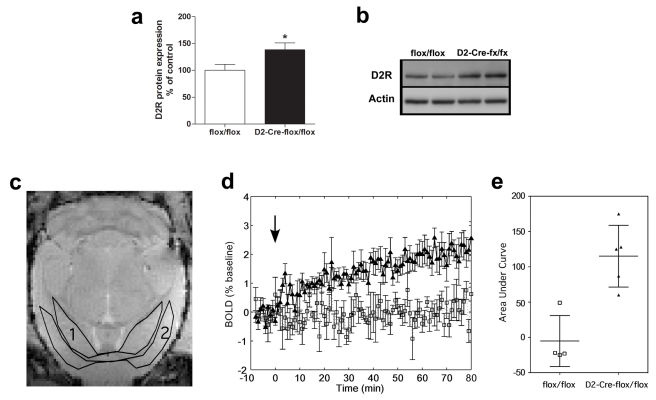
Increased dopamine receptor expression and activity in VAChT^D2-Cre-flox/flox^ mice. (a) Striatal D2R protein levels inVAChT^D2-Cre-flox/flox^ mice and controls (*n* = 8). * *p*<0.05. (b) D2R representative immunoblot blot. (c) Axial MRI FLASH image (500 μm thick) through the striatum (outlined region 1) and cerebral cortex (outlined region 2). (d) Average BOLD signal change in the striatum relative to cerebral cortex prior to and following injection of SFK 81297 at time zero (black arrow). Signal response for VAChT^D2-Cre-flox/flox^ mice (*N* = 5) is shown with black triangles, and response for control mice (*N* = 4) is shown with open squares. Error bars represent the standard error of the mean. (e) Area under the curve for VAChT^D2-Cre-flox/flox^ mice (*N* = 5) is shown with black triangles, and response for control mice (*N* = 4) is shown with open squares. The difference between genotypes was statistically significant (*p*<0.01).

To confirm the increased alteration of dopamine receptors in the striatum of VAChT^D2-Cre-flox/flox^ mice we initially performed Western blots. Unfortunately, we were unable to obtain a reliable D1 antibody that showed specific detection of D1R (unpublished data). However, we obtained a D2 antibody that labelled only one major band with the correct molecular mass (Figure 9a). Quantification of immunoblots confirmed increased expression of D2R (Figure 9a,b, *t*_(14)_ = −3.628, *p*<0.01). In order to provide an independent measure of D1R activity and test if D1-mediated responses would be altered in VAChT-eliminated mice, we used pharmacological magnetic resonance imaging (*ph*MRI) [51],[52]. *ph*MRI is a variant of functional magnetic resonance imaging that indirectly detects neuronal activity using blood oxygenation level-dependent (BOLD) MRI signal changes [53] to detect functional effects of pharmacological agents in intact systems in vivo with high temporal and spatial resolution. A 9.4T anatomic MRI of the mouse brain (Figure 9c) was used to outline regions of interest in the striatum and cortex. The average difference in BOLD effect between striatum and cortex (Figure 9d) indicates that there is increased neuronal activation in the striatum in VAChT^D2-Cre-flox/flox^ mice following injection of SFK 81297 (3 mg/kg, Figure 9d). The change in the BOLD response after administration of the selective D1R agonist SKF 81297 relative to baseline (prior to injection) was then compared between the two genotypes. Saline administration prior to SKF 81297 did not alter BOLD signal (unpublished data). In contrast, injection of SKF 81297 lead to a slow increase in striatal BOLD response (area under the curve) in VAChT^D2-Cre-flox/flox^ mice compared to control mice following injection of the D1R agonist (Figure 9d and e, *p*<0.01).

**Figure 10:**
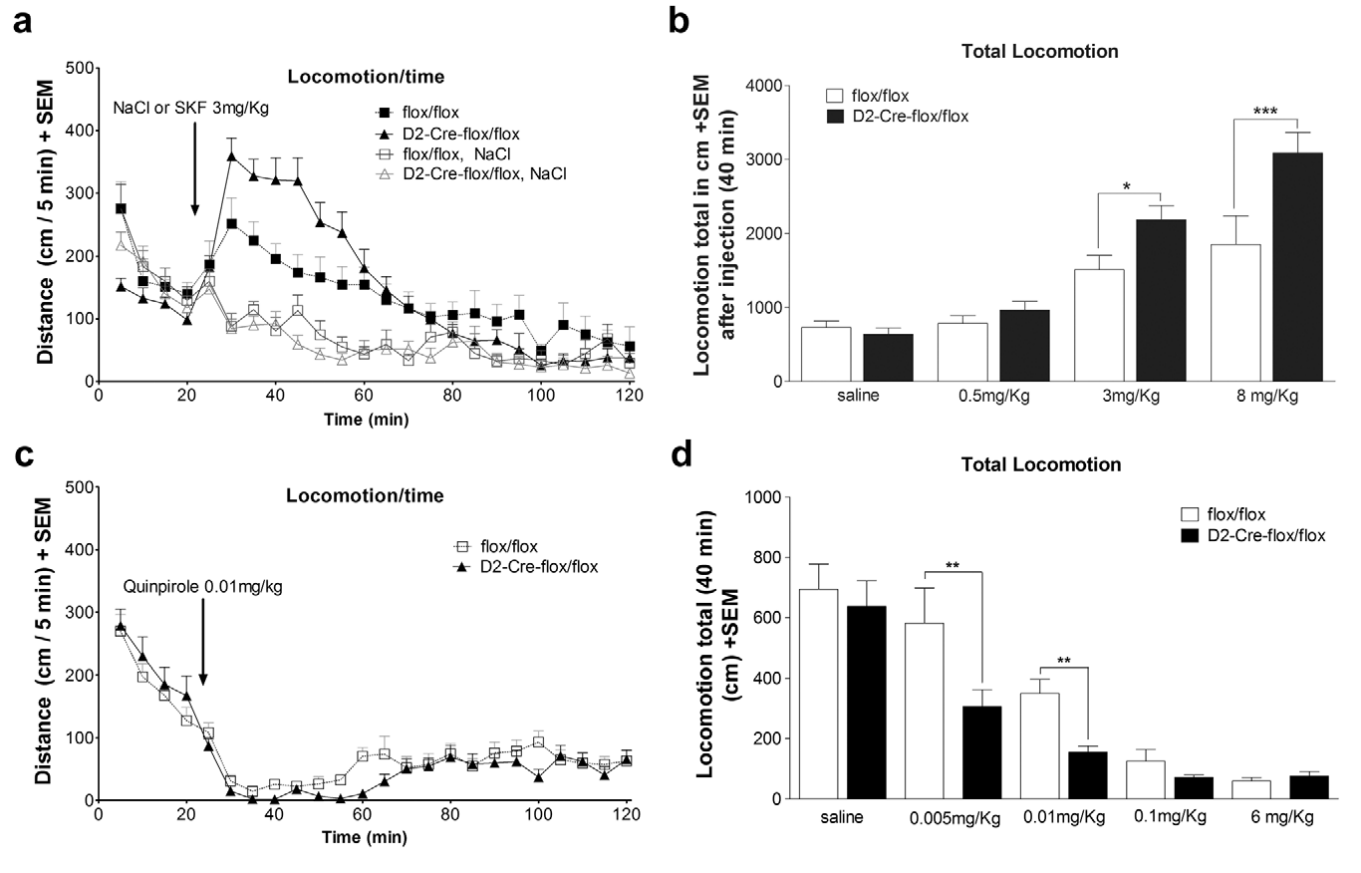
Effect of D1R or D2R agonists on locomotor activity in VAChT^D2-Cre-flox/flox^ mice. (a) Effect of injection of SFK 81297 (3 mg/kg) 20 min after the mice were introduced to the open field, (b) dose-response for SKF 81297, (c) effect of quinpirole (0.01 mg/kg) as in (a), and (d) dose response for quinpirole. * *p*<0.05, ** *p*<0.01, and *** *p*<0.001. *N* = 10 and 13 for saline, SKF 81297 *N* = 7 and 5 for 0.5 mg/kg, *N* = 9 and 4 for 3 mg/kg, and *N* = 7 and 9 for 8 mg/kg. For quinpirole *N* = 7 and 13 for 0.005 mg/kg, *N* = 17 and 15 for 0.01 mg/kg, *N* = 11 and 12 for 0.1 mg/kg, and *N* = 11 and 13 for 6 mg/kg for control and VAChT^D2-Cre-flox/flox^ mice, respectively.

To test if the increased expression/sensitivity of D1R and D2R has direct behavioural consequences, we investigated the effects of the selective dopaminergic agonists SKF 81297 (D1R agonist) and quinpirole (D2R agonist) on locomotor activity. VAChT^D2-Cre-flox/flox^ mice had significantly higher locomotor responses to two doses of SKF 81297 (Figure 10a and b, two-factor ANOVAs revealed significant effect of genotype, *F*_(1,66)_ = 11.654, *p*<0.01, significant effect of drug concentration, *F*_(3,66)_ = 34.476, *p*<0.001, and significant Drug Concentration × Genotype interaction, *F*_(3,66)_ =  4.277, *p*<0.01, Tukey post hoc test showed significant differences with SKF 81297 doses of 3 mg/kg (*p*<0.01) and 8 mg/kg (*p*<0.001)). Moreover, VAChT^D2-Cre-flox/flox^ mice also showed enhanced inhibition of locomotion in response to low doses of the D2R-selective agonist quinpirole (Figure 10c and d, ANOVA showed significant effect of genotype, *F*_(1,111)_ = 12.543, *p*<0.001, significant effect of drug, *F*_(4,111)_ = 42.223, *p*<0.001, but the interaction was not significant, *F*_(4,111)_ = 2.052, *p* = 0.092). Analysis of locomotion in response to the individual doses showed a significant difference for 0.005 and for 0.01 mg/kg quinpirole dose (*p*<0.01). Taken together, these data reveal important alterations in the expression and function of striatal dopamine receptors in VAChT^D2-Cre-flox/flox^ mice.

## Discussion

Here we present a series of evidence that delineates the role of released ACh from those of VGLUT3-dependent glutamate release from striatal cholinergic interneurons. Our data provide a novel perspective on the function of striatal cholinergic neurons suggesting the possibility that they can use distinct neurotransmitters to regulate striatal circuitry. We found that elimination of VAChT in the striatum, without disruption of VGLUT3, did not cause overt disruptions or alterations in several behavioural tasks previously thought to be dependent on striatal ACh release, such as motor learning, sensorimotor gating, and spontaneous locomotor activity. However, we uncovered a novel form of regulation of MSNs by cholinergic tone, and found that selective silencing of striatal ACh release results in an increase in the responses to D1R and D2R agonists. In contrast to the effects of direct dopamine receptor agonists, we found that overall these mice do not show increased locomotor response to cocaine. Similarly, sensitization and rewarding effects of cocaine did not seem to be dependent on striatal release of ACh. Thus, our results significantly depart from previous studies in which the specific contributions of striatal ACh release (mediated by VAChT) were not separated from those of glutamate release (mediated by VGLUT3). These data suggest that VGLUT-3 dependent glutamate release may influence locomotor activity and responses to cocaine considerably more than VAChT-dependent ACh release. Our data suggest that targeted approaches aimed at inhibiting VAChT activity in the striatum may potentially provide a novel strategy to enhance dopaminergic signalling, without causing other major behavioural disturbances.

### VAChT^D2-cre-flox/flox^ Mice Have Normal Motor Performance, Sensorimotor Gating, and Motor Learning

Our studies in VAChT^D2-cre-flox/flox^ mice indicated that elimination of ACh release in the striatum does not seem to play a major role in motor function and motor learning, at least for acrobatic motor skills in the rotarod test. This observation is also in agreement with previous experiments in striatal cholinergic neuron-ablated mice that presented no deficiency in rotarod performance [31]. However, we cannot completely exclude more subtle effects of ACh in fine motor tuning and motor tasks. For example, the chronic nature of elimination of ACh release in our experiments may lead to adaptations in motor behaviour. Future experiments using VAChT^D2-Cre-flox/flox^ mice and more sophisticated motor behavioural tests may be necessary to pinpoint possible roles for striatal ACh in motor learning and performance.

There are multiple lines of evidence that pharmacological modulation of cholinergic receptors regulates locomotor activity. It is known that muscarinic antagonists increase locomotor activity and M1 and M4 muscarinic receptor KO mice are hyperactive [54]–[57]. Moreover, we have recently observed that mice with a significant decrease in VAChT expression in the whole forebrain show hyperactivity [32]. The present work provides compelling evidence for more selective roles of the neurotransmitter ACh in the striatum, indicating that decreased striatal expression of VAChT does not cause overt motor consequences. These results may be of particular importance, since there have been reports that in Huntington's disease VAChT levels are decreased in the striatum [58]. Our data suggest, however, that this alteration is unlikely to contribute to gross motor symptoms observed in Huntington's disease. Cholinergic neurotransmission in brain regions other than the striatum may still play a role in control of locomotion.

Previous attempts to assess the function of cholinergic neurons in the striatum were performed following the ablation of cholinergic neurons using immunotoxin-mediated cell targeting. Injection of toxin targeting transgenic cholinergic neuron in the accumbens led to an 80% decrease in ChAT-positive neurons [30]. Elimination of cholinergic neurons in the accumbens by this means inhibited certain forms of reward-related learning; however, it also induced hyperactivity and increased sensitivity to the locomotor and the rewarding effects of cocaine, including increased sensitivity in the CPP test to low doses of cocaine [28],[29],[31]. In contrast, recent experiments using an optogenetic approach to inactivate or activate cholinergic neurons in the accumbens found no effects of inactivation of these neurons on locomotor activity, albeit their silencing prevented the response to cocaine in a CPP test [44]. Thus, elimination of cholinergic neurons in the accumbens seemed to increase sensitivity to cocaine-induced CPP [29], whereas optogenetics silencing of these neurons blocked cocaine-induced CPP [44]. The reason for the different outcome in these two experiments is not entirely clear at the moment, but could be related to the chronic versus acute nature of the manipulations. Although in our experiments we have targeted the whole striatum, rather than only the accumbens, we did not detect major alterations in cocaine-induced CPP, suggesting that the above effects obtained with neuronal ablation or by optogenetics manipulation may be linked not to loss of cholinergic transmission per se but rather to suppression of glutamate release from cholinergic neurons.

While an optogenetic approach provides a novel paradigm to acutely activate or inactivate populations of neurons, it is unlikely that this method can separate VAChT from VGLUT3-dependent neurotransmission as selectively as that which can be achieved using VAChT^D2-Cre-flox/flox^ mice. Interestingly, recent data have shown that cholinergic neurons in the habenula secrete both ACh and glutamate (mediated by VGLUT1), and release of either of these neurotransmitters appears to depend on the frequency of stimulation [22]. Basal forebrain neurons in culture release both ACh and glutamate [59]. Importantly, recent work shows that optogenetics stimulation of striatal cholinergic neurons can evoke synaptic glutamatergic neurotransmission onto MSNs, with predominant activity over NMDA receptors [27]. The co-release of glutamate with dopamine has also been described [60],[61], suggesting that interpretation of the roles of dopaminergic neurons will also need to take into account glutamate co-release. Therefore, the co-release of glutamate with classical neurotransmitters may be a more common mechanism than previously appreciated and may have a broad impact in circuitry control. However, we cannot discard the possibility that other neuromodulators released from cholinergic neurons, such as ATP or peptides, could also play a role as co-transmitters.

The role of VGLUT3 in striatal function is far from being fully understood [62]. Interestingly, with respect to striatum-related behaviour, VGLUT3-null mice show hyperactivity and increased response to the locomotor effects of cocaine [25]. Therefore, mice lacking VGLUT3 show a phenotype that is remarkably similar to that of mice in which cholinergic neurons in the accumbens were targeted by an immunotoxin [29],[30]. Experiments in VGLUT3-null mice suggested that the absence of VGLUT3 causes a decrease in striatal cholinergic tone. VGLUT3 is used by the striatal vesicles to facilitate VAChT-mediated ACh storage in synaptic vesicles [25],[62]. However, measurements of ACh release in VGLUT3-null mice have indicated only a modest reduction, by 30% to 40% [25], compared to almost 100% inhibition in VAChT^D2-Cre-flox/flox^ mice. It is unlikely that 40% reduction in ACh release observed in VGLUT3-null mice can be responsible for the hyperactive phenotype. Indeed, independent mouse lines with a 50% decrease in VAChT expression, and concomitant reduction of ACh release [16],[36],[63], did not present increased locomotor activity in the open field [16],[35]. We conclude that the locomotor phenotypes observed previously in striatal cholinergic neuron-ablated mice [29],[31] and in VGLUT3-null mice [25] are either a consequence of the disruption of VGLUT3-mediated neurotransmission or the combination of reducing both glutamatergic and cholinergic activity simultaneously from these neurons. Future experiments using VAChT^D2-Cre-flox/flox^ mice, VGLUT3 floxed mice, and double knockouts will be necessary to provide an assessment of independent effects of VGLUT3-mediated neurotransmission in the striatum.

Although we have focused on striatal-related behaviours, the extent by which alterations in VAChT expression in other brain regions in VAChT^D2-cre-flox/flox^ mice may contribute to these phenotypes should also be taken into account. We did not detect Cre-expression in cholinergic neurons in the penduculopontine area, for example (Figure S2), which harbours groups of cholinergic neurons that project to the midbrain and thalamus and could influence striatal function. However, we cannot completely exclude the possibility that cholinergic neurons in other brain regions would not be targeted in our mouse line. At the same time, as the phenotypes described here seem to be mainly striatal specific and cholinergic interneurons provide the almost exclusive source of cholinergic tone in the striatum, it is unlikely that other groups of cholinergic neurons would have contributed to the observed behaviours.

Elimination of cholinergic neurotransmission in the striatum did not cause hyperlocomotion, however the responses to direct activation of dopamine receptors were substantially increased. Both behavioural and *ph*MRI analysis indicated an increased response to D1R agonist. Western blot analysis also showed selective increase of D2R expression in the striatum. Moreover, in addition to the increased D2R levels in the striatum, which likely reflect a combination of pre- and post-synaptic receptors, we also uncovered increased D2-like receptor pre-synaptic activity, revealed by the increased sensitivity of VAChT^D2-Cre-flox/flox^ mice to low doses of quinpirole. Certainly, we cannot rule out that changes at the level of receptors play a more complex role in regulating locomotor activity in VAChT^D2-Cre-flox/flox^ mice. Indeed, GPCRs may have agonist-independent activity [64],[65]. The locomotor effects of cocaine seem to depend mainly on inhibition of the dopamine transporter [66]. However, acetylcholine can affect release of dopamine via distinct nicotinic receptors [19], as well as regulate both dopamine release and activity of MSNs, via distinct muscarinic receptors [56],[57],[67]. The fact that both D1R and D2R had increased expression in the striatum would suggest that VAChT^D2-Cre-flox/flox^ mice should be more responsive to dopamine and might present increased spontaneous locomotor activity or cocaine-induced locomotion or CPP. However, this was not the case. It is likely that cell-autonomous compensatory mechanisms related to disrupted cholinergic function significantly altered striatal circuitry, preventing such a simple relationship. For example, because M4 muscarinic receptors seem to specifically regulate D1R-mediated signalling [56],[57],[68], it is possible that the increased expression of M4 receptors we detected in the striatum could counterbalance D1R-mediated responses in vivo, leading to unaltered locomotor activity. Moreover, because D2-like pre-synaptic receptors may be more active in VAChT^D2-Cre-flox/flox^ mice, elimination of ACh release in the striatum may also affect pre-synaptic control of dopamine release. The slightly decreased turnover of dopamine in mice without striatal VAChT supports the notion of direct consequences of reduced cholinergic tone at the level of dopaminergic terminals. Thus, behavioural analysis of VAChT^D2-Cre-flox/flox^ mice indicates that control of locomotor function and response to cocaine mediated by dopamine might become more complex in the absence of cholinergic tone. Future experiments will be needed to evaluate direct consequences of elimination of either acetylcholine or glutamate neurotransmission originating from striatal cholinergic neurons on dopamine transmission.

### Conclusion

The present data provide direct and indirect evidence that striatal cholinergic neurons can use two different neurotransmitters to regulate striatal function. Hence, re-evaluation of previously attributed functions of striatal cholinergic tone is warranted. The data indicate that VGLUT3-mediated glutamatergic neurotransmission originating from cholinergic neurons may have greater influence on striatal function than previously envisioned. The behavioural consequences of selective elimination of VAChT, and thus cholinergic transmission, in the striatum are remarkably minimal, at least for the locomotion control by the striatal complex. One intriguing phenotype uncovered in mutant mice is an increase in dopamine receptors' expression and function without major alterations in cocaine-induced behaviours. Our experiments provide evidence that targeting VAChT in the striatum can up-regulate dopamine receptors and thus could be used in conditions of dopamine deficiency and abnormally increased cholinergic activity, as found in individuals with PD.

## Materials and Methods

### Animals

The isolation of a VAChT genomic clone has been described previously [36]. The genomic clone was used to construct a gene-targeting vector in which we added LoxP sequences flanking the VAChT open reading frame and a TK-Neo cassette. Generation of VAChT^flox/flox^ mice is described elsewhere [32], and the construct is shown in Figure S2. Briefly, after removal of the TK-Neo cassette, one LoxP sequence was present 260 bp upstream from the VAChT translational initiation codon, and a second LoxP sequence was located approximately 1.5 kb downstream from the VAChT stop codon and within the second ChAT intron. Note that this vector is distinct from that previously used for generation of VAChT KD mice [36].

D2-Cre mice (Drd2, Line ER44) were obtained from the GENSAT project via the mutant mouse regional resource centers. VAChT^D2-Cre-flox/flox^ mice were generated by crossing VAChT^flox/flox^ with the D2-Cre mouse line. We then inter-crossed VAChT^D2-Cre-flox/wt^ to obtain VAChT^D2-Cre-flox/flox^ mice. Because these mice were apparently normal and fertile, we bred VAChT^D2-Cre-flox/flox^ mice and VAChT^flox/flox^ to obtain all the mice used in the present study. These mice were backcrossed to C57BL/6J mice for five generations. Unless otherwise stated, all control mice used were VAChT^flox/flox^ littermate mice without the Cre transgene.

After the completion of this work we were made aware that the BAC used to generate D2-Cre mice carried an extra gene, *ttc2*, and a recent report suggests that homozygous D2-GFP mice, generated using the same BAC construct, are hyperactive and show a number of dopamine-related phenotypes [50]. However, as these authors point out, their experiments cannot discern if the phenotypes uncovered are due to the BAC positioning insertion or to the extra copy of *ttc2.* We confirmed that the D2-Cre indeed have increased expression of the TTC2 mRNA (unpublished data). However, heterozygous D2-Cre mice showed no locomotor phenotype. Moreover, these mice showed normal levels of D1R, D2R, and M4-muscarinic receptors (Figure S6). Hence, neither the phenotypes nor the molecular changes observed in VAChT^D2-Cre-flox/flox^ mice are due to the BAC transgene.

Rosa26-YFP mice (B6.129X1-Gt(ROSA)26Sor^tm1(EYFP)Cos/J^, stock number 006148) were obtained from Jackson Laboratories. Animals were housed in groups of three to four mice per cage without environment enrichment in a temperature-controlled room with 12-h light–12-h dark cycles, and food and water were provided ad libitum. Mouse stocks were SPF, however experimental subjects were kept in a conventional mouse facility.

### Ethics Statement

All studies were conducted in accordance with the NIH and the Canadian Council of Animal Care (CCAC) guidelines for the care and use of animals with approved animal protocol from the Institutional Animal Care and Use Committees at the University of Western Ontario (protocol number 2008–089). Only male mice were used for the behavioural studies, and they were at least 12 weeks old. Mice were randomly assigned to distinct experimental groups. Only mice used for evaluation of spontaneous locomotor behaviour were used in other tasks.

### Immunofluorescence, qPCR, and Western Blot

For the immunofluorescence experiments we followed a protocol previously described [35],[69]. For mRNA analyses tissue samples were frozen in a mixture of dry ice/ethanol and kept at −80°C until used as described [70]. Immunoblotting was performed as described elsewhere [36],[71],[72].

### Measurements of [^3^H]ACh Release from Brain Slices

Slices were obtained from the striatum and hippocampus of control and test mice, labelled with [^3^H]methyl-choline, and the release of labelled ACh was determined essentially as described [73] except that 33 mM KCl was used as a depolarizing stimuli.

### Preparation of Synaptosomes and Glutamate Release

Striatal synaptosomes were prepared by the method of [74],[75] as previously described [76]. Glutamate release was followed continuously using a fluorimetric method [77] exactly as previously described [78].

### Measurements of Locomotor Activity

All behavioural experiments were performed between 9 a.m. and 4 p.m. in the light cycle, essentially as previously described [16],[35] except the spontaneous activity of the first hours of the dark cycle was done from 7 p.m. to 10 p.m.

### Catecholamine Measurement

The dissected brain tissues were homogenized in 0.2 M perchloric acid with 100 µM EDTA-2Na. Samples were spun in a microcentrifuge at 12,000 rpm for 15 min at 4°C. Samples of the supernatant were then analyzed for norepinephrine (NE), dopamine (DA), and its two metabolites, 3,4-dihydroxyphenylacetic acid (DOPAC) and homovanillic acid (HVA) by NoAb BioDiscoveries (Mississauga, ON).

The HPLC used was an Eicom EP-700 with electrochemical detection (Eicom ECD-700). To elute catecholamines from the reverse phase column (3.0×100 mm SC-3ODS column, Eicom), a mobile phase consisting of 0.1 M citric acetate buffer pH 3.5 with 5 mg/ml EDTA-2Na, 220 mg/L sodium octane sulfonate, and 22% methanol was used.

### Acoustic Startle Measurements

These experiments have been described in [79]. Briefly, animals were acclimatized 3–5 times to the startle boxes (Med Associates). Habituation of startle was measured using 30 startle pulses (20 ms, white noise, 115 dB on a 65 bd white noise background) with an inter-trial interval of 20 s. Subsequently, prepulse inhibition was measured by displaying 50 startle stimuli with either no prepulse (pulse alone), a 75 dB (4 ms white noise) prepulse preceding the pulse by either 30 ms or 100 ms, or a 85 db prepulse (30 ms or 100 ms interval). Each of the five trial types were displayed 10 times in a pseudorandomized order. PPI is expressed as the average startle response to the respective prepulse trials in relation to the pulse alone trials.

### Grip-Force and Wire-Hang

A Grip Strength Meter from Columbus Instruments (Columbus, OH) was used to measure forelimb grip strength essentially as described [36]. For the wire-hang test each mouse was placed on a metal wire-grid, which was slowly inverted and suspended 40 cm above a piece of foam as previously described [36]. The time it took for each mouse to fall from the cage top was recorded with a 60 s cut-off.

### Rotarod

The rotarod task followed a previously described protocol [36].

### CPP

The CPP protocol was modified from [80]. Briefly, CPP was performed in a three chamber apparatus containing two large compartments with differences in visual and tactile cues, separated by a neutral area. In day 1 (habituation), mice were placed in the central compartment and allowed free access to the entire apparatus for 30 min. The time spent in each compartment was recorded. On days 2–7 (the conditioning phase), mice received alternating injections of cocaine or vehicle and were immediately confined into one of the two large compartments for 30 min. A combination of unbiased and biased allocation was used. On day 8 (test day) mice were once again allowed free access to all three compartments for 30 min, and the time spent in each compartment was recorded. For the CPP extinction and reinstatement, the protocol previously described [80] was followed.

### Behavioural Sensitization

Behavioural sensitization was performed as described [57].

### Object Recognition Memory

The general procedure was previously described, but for analysis we used Anymaze [35].

### Pharmacological Magnetic Resonance Imaging (*ph*MRI)

Mice (VAChT^D2-cre/flox/flox^, *N* = 5; control, *N* = 4) were anesthetized with 4% isofluorane and maintained at 1.5% isoflurane during the MRI scanning. Two intraperitoneal (I.P.) catheters (26 gauge, Abbotcath) were used for injection of saline and SFK. The catheters were secured in place with subcutaneous sutures. Catheters were connected to polyethelyne tubing (PE50, VWR, Canada) and to syringes containing saline and SFK for remote injection during imaging. Mice were placed in a custom built frame designed to secure the skull and minimize respiration induced movement during image acquisition. Mice were imaged on a 9.4 Tesla small animal MRI scanner (Agilent, Palo Alto, CA) equipped with a two-channel surface coil (diameter  = 2 cm). A fast low angle shot (FLASH) pulse sequence was used to acquire anatomical images (field of view  = 19.2×19.2 mm^2^, matrix  = 128×128, repetition time  = 50 ms, echo time  = 11 ms, flip angle  = 11°, and 10 averages). Respiratory gated lower resolution FLASH images were also acquired for pharmacological imaging (field of view  = 19.2×19.2 mm^2^, data matrix  = 64×64, repetition time  = 15 ms, echo time  = 7 ms, flip angle  = 11°, and 1 average) to measure blood oxygen level–dependent (BOLD) signal changes. Seven contiguous axial slices (500 μm thick) covered the brain. Each animal received two injections: first, an injection of 0.5 ml physiological saline (0.9%) administered over a 30 s period (control), and second, SFK 81297 (3 mg/kg), diluted in 0.5 ml physiological saline, also administered over a 30 s period (drug). For the control experiment, images were acquired for 8 min prior to saline injection and then for 20–50 min following injection. For the drug experiment, images were acquired for 8 min prior to drug injection and then for 80–180 min after injection. Throughout the imaging session, body temperature and respiration rate was monitored every 10 min using the MR-Compatible, Model 1025 monitoring system (Small Animal Instruments Inc., Stony Brook, NY). Temperature was maintained at 37.5°C using a warm air blower, and respiration rate ranged from 45–66 (mean 54 BPM). Following imaging, mice were euthanized by cervical dislocation while still under isoflurane anaesthesia.

To limit the influence of global motion on the functional result, the signal intensity difference between striatum and cortex was used to examine the effect of SFK 81297 on the striatum as a function of time. A single slice transecting the striatum was chosen for analysis in each animal (Figure 9c). BOLD signal change was expressed as the percentage change relative to the average baseline signal (first 50 images) prior to drug injection.

### Statistical Analysis

Data are expressed as mean ± SEM. Sigmastat 3.1 software was used for statistical analysis. Comparison between two experimental groups was done by Student's *t* test or Mann-Whitney Rank Sum Test when the data did not follow a normal distribution. When several experimental groups were analyzed, we used two-way analysis of variance (ANOVA). For locomotion experiments we used ANOVA with repeated measures, and when appropriate, a Tukey post hoc comparison test was used. For pharmacological MRI, the area under the curve of the signal time course was compared between VAChT^D2-cre/flox/flox^ mice and control mice using a Student's *t* test.

## Supporting Information

Figure S1 D2-Cre drives the expression of Cre in striatal cholinergic neurons. (a) Sections from different regions of the central nervous system were immunostained for CHT1 (Red) and YFP (Green) in D2-Cre;Rosa26-YFP mice. Arrows show localization of Cre expression (YFP) in cholinergic neurons (CHT1 staining). Arrowheads show cholinergic neurons that do not express Cre.
(TIF)

Figure S2 Representation of VAChT^D2-Cre-flox/flox^ allele and identification by genotyping. (a) Cartoon representing the VAChT alleles before and after recombination. (b) PCR genotyping for VAChT^flox/flox^ and VAChT^D2-Cre-flox/flox^ mice. Lanes 1 and 2 WT PCR product, lane 3 flox/flox PCR product, and lane 4 heterozygous mice PCR product. (c) Lane 1 VAChT^flox/flox^, lane 2 VAChT^D2-Cre-flox/flox^, lane 3 VAChT^D2-Cre-flox/flox^, and lane 4 VAChT^flox/flox^ genotyping.
(TIF)

Figure S3 Cholinergic parameters in the spinal cord of VAChT^D2-Cre-flox/flox^ mice. (a, b, c) Quantification of mRNA expression for VAChT, ChAT, and CHT1, respectively, in the Spinal cord. (d, e, f) Quantification of protein levels in the Spinal cord for VAChT, ChAT, and CHT1, respectively. Synaptophysin immunoreactivity was used to correct for protein loading between experiments. (g) Representative Western blot of VAChT, synaptophysin, ChAT, CHT1, and actin ** *p*<0.01 and *** *p*<0.001.
(TIF)

Figure S4 Motor function is not altered in VAChT^D2-Cre-flox/flox^ mice. (a) Grip-force analysis of VAChT^flox/flox^ and VAChT^D2-Cre-flox/flox^ mice. (b) Time spent hanging upside-down from a grid, to measure fatigue for VAChT^flox/flox^ mice and VAChT^D2-Cre-flox/flox^ mice (cut-off time 60 s). (c) Motor learning and acrobatic motor skills of VAChT^D2-Cre-flox/flox^ mice determined using the rotarod (flox/flox, *N* = 14; D2-Cre-flox/flox, *N* = 17). (d) Object recognition memory in VAChT^flox/flox^ and VAChT^D2-Cre-flox/flox^ mice. * *p*<0.05.
(TIF)

Figure S5 Sensorimotor gating is not altered in VAChT^D2-Cre-flox/flox^ mice. (a) Short-term habituation of acoustic startle responses to 30 acoustic startle stimuli of 115 db white noise delivered every 20 s. (b) Prepulse inhibition of acoustic startle responses with different prepulse intensities and prepulse-pulse intervals as indicated.
(TIF)

Figure S6 Biochemical and behavioural parameters of heterozygous D2-Cre mice. (a) Horizontal activity as described in Figure 4. (b) Total ambulance during 2 h. (c) Rearing during 2 h. (d) D1R mRNA, (e) D2R mRNA, an (f) mAChR-4 mRNA levels.
(TIF)

Table S1 Percentage of co-localization between cell with Cre-induced recombination and CHT1.
(TIF)

## Abbreviations

BOLD: blood oxygenation level-dependent
ChAT: choline acetyltransferase
CHT1: high-affintiy choline transporter
CPP: conditioned place preference
*ph*MRI: pharmacological MRI
PPI: pre-pulse inhibition
VAChT: vesicular acetylcholine transporter
VGLUT3: vesicular glutamate transporter 3
