# Supplementary figures and images for: Elimination of the Vesicular Acetylcholine Transporter in the Striatum Reveals Regulation of Behaviour by Cholinergic-Glutamatergic Co-Transmission
Source: PLoS Biol. 2011 Nov 8;9(11):e1001194. doi: 10.1371/journal.pbio.1001194 (PMC3210783; doi:10.1371/journal.pbio.1001194)

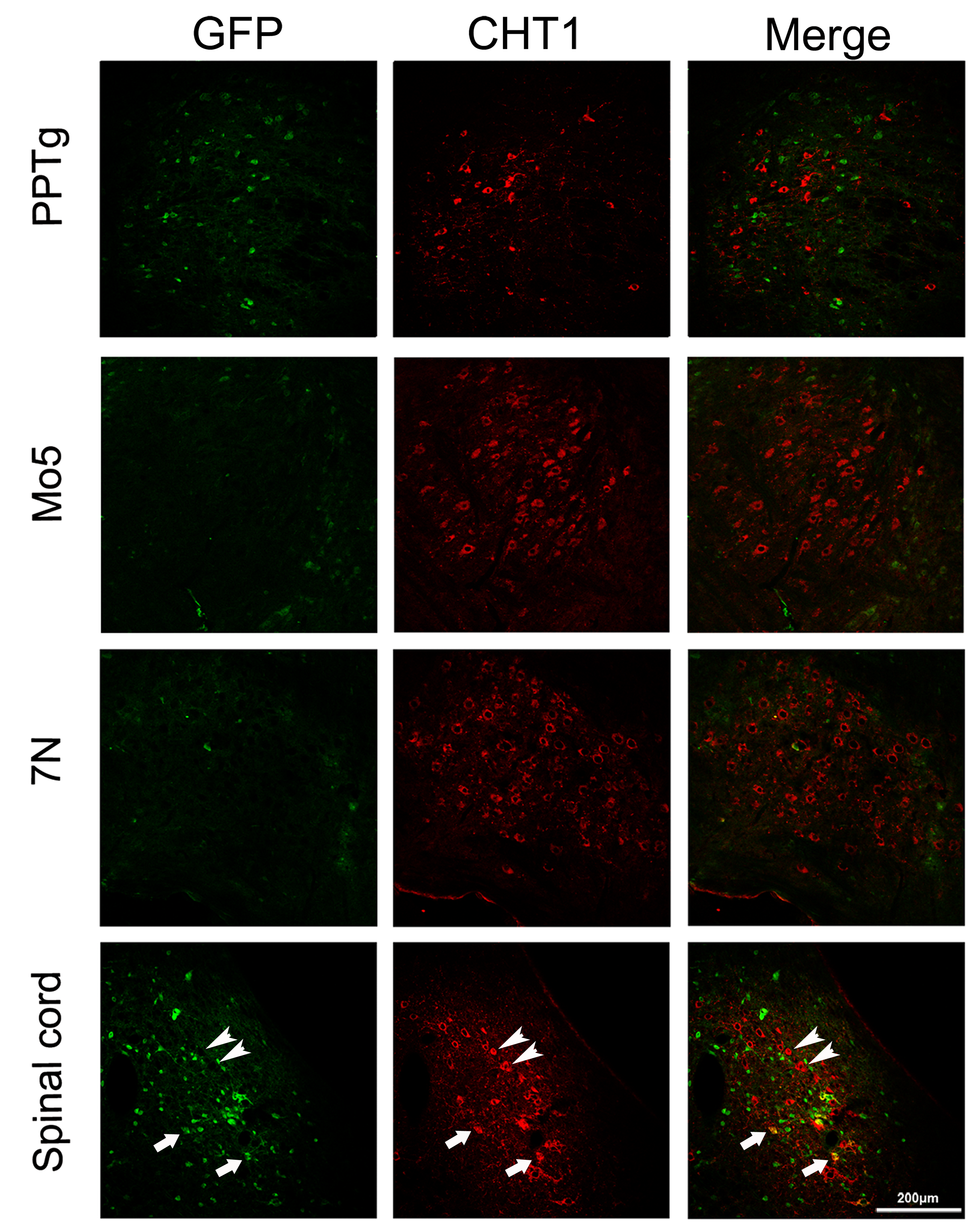

Supplement: Figure S1 — D2-Cre drives the expression of Cre in striatal cholinergic neurons. (a) Sections from different regions of the central nervous system were immunostained for CHT1 (Red) and YFP (Green) in D2-Cre;Rosa26-YFP mice. Arrows show localization of Cre expression (YFP) in cholinergic neurons (CHT1 staining). Arrowheads show cholinergic neurons that do not express Cre. (TIF) [file pbio.1001194.s001.tif]

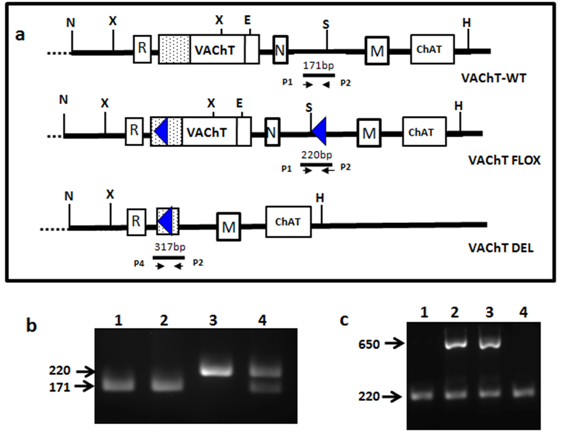

Supplement: Figure S2 — Representation of VAChTD2-Cre-flox/flox allele and identification by genotyping. (a) Cartoon representing the VAChT alleles before and after recombination. (b) PCR genotyping for VAChTflox/flox and VAChTD2-Cre-flox/flox mice. Lanes 1 and 2 WT PCR product, lane 3 flox/flox PCR product, and lane 4 heterozygous mice PCR product. (c) Lane 1 VAChTflox/flox, lane 2 VAChTD2-Cre-flox/flox, lane 3 VAChTD2-Cre-flox/flox, and lane 4 VAChTflox/flox genotyping. (TIF) [file pbio.1001194.s002.tif]

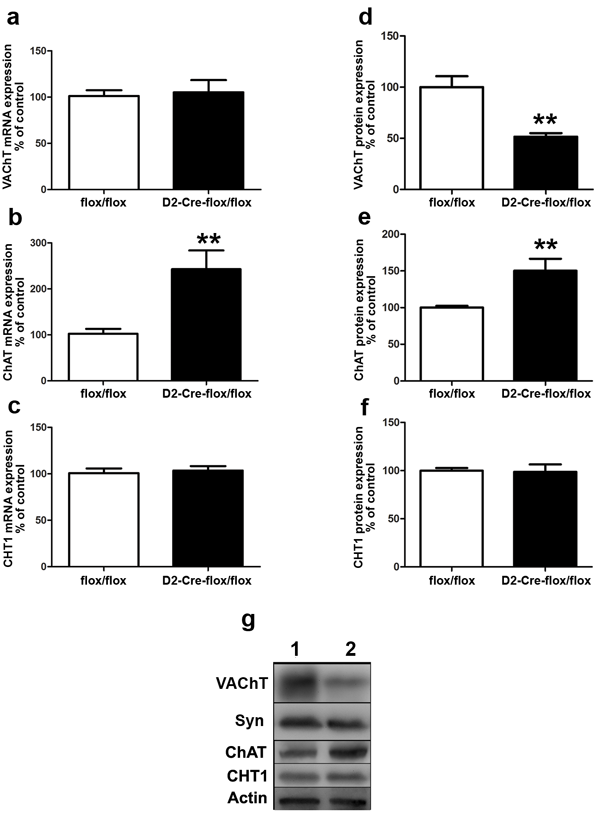

Supplement: Figure S3 — Cholinergic parameters in the spinal cord of VAChTD2-Cre-flox/flox mice. (a, b, c) Quantification of mRNA expression for VAChT, ChAT, and CHT1, respectively, in the Spinal cord. (d, e, f) Quantification of protein levels in the Spinal cord for VAChT, ChAT, and CHT1, respectively. Synaptophysin immunoreactivity was used to correct for protein loading between experiments. (g) Representative Western blot of VAChT, synaptophysin, ChAT, CHT1, and actin ** p<0.01 and *** p<0.001. (TIF) [file pbio.1001194.s003.tif]

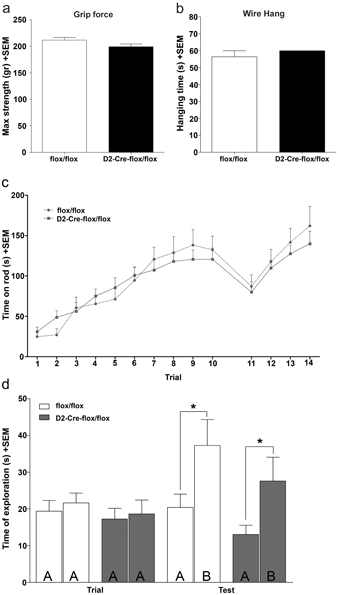

Supplement: Figure S4 — Motor function is not altered in VAChTD2-Cre-flox/flox mice. (a) Grip-force analysis of VAChTflox/flox and VAChTD2-Cre-flox/flox mice. (b) Time spent hanging upside-down from a grid, to measure fatigue for VAChTflox/flox mice and VAChTD2-Cre-flox/flox mice (cut-off time 60 s). (c) Motor learning and acrobatic motor skills of VAChTD2-Cre-flox/flox mice determined using the rotarod (flox/flox, N = 14; D2-Cre-flox/flox, N = 17). (d) Object recognition memory in VAChTflox/flox and VAChTD2-Cre-flox/flox mice. * p<0.05. (TIF) [file pbio.1001194.s004.tif]

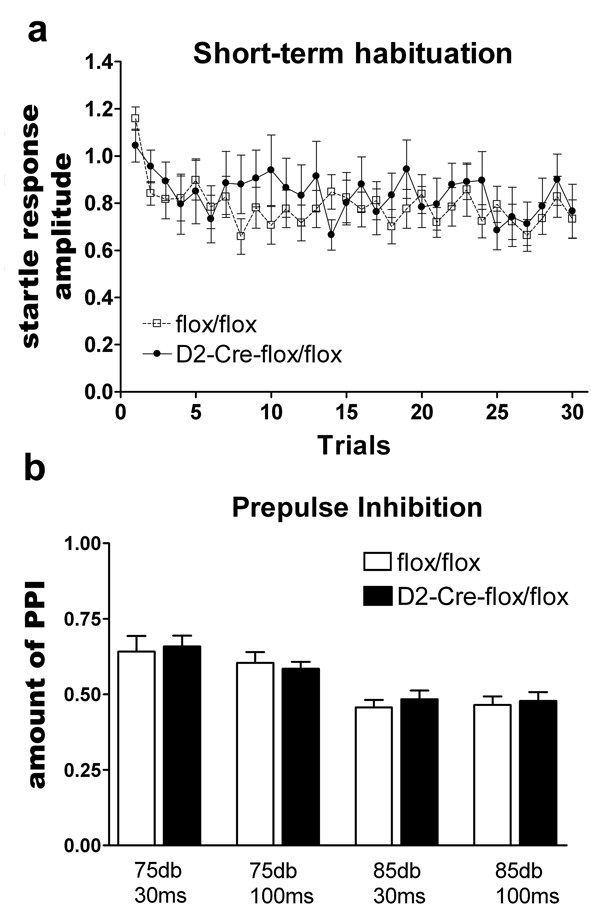

Supplement: Figure S5 — Sensorimotor gating is not altered in VAChTD2-Cre-flox/flox mice. (a) Short-term habituation of acoustic startle responses to 30 acoustic startle stimuli of 115 db white noise delivered every 20 s. (b) Prepulse inhibition of acoustic startle responses with different prepulse intensities and prepulse-pulse intervals as indicated. (TIF) [file pbio.1001194.s005.tif]

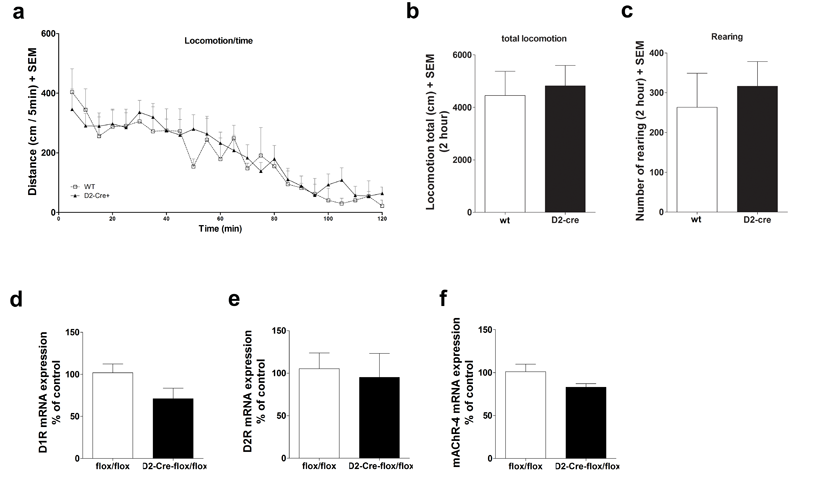

Supplement: Figure S6 — Biochemical and behavioural parameters of heterozygous D2-Cre mice. (a) Horizontal activity as described in Figure 4. (b) Total ambulance during 2 h. (c) Rearing during 2 h. (d) D1R mRNA, (e) D2R mRNA, an (f) mAChR-4 mRNA levels. (TIF) [file pbio.1001194.s006.tif]

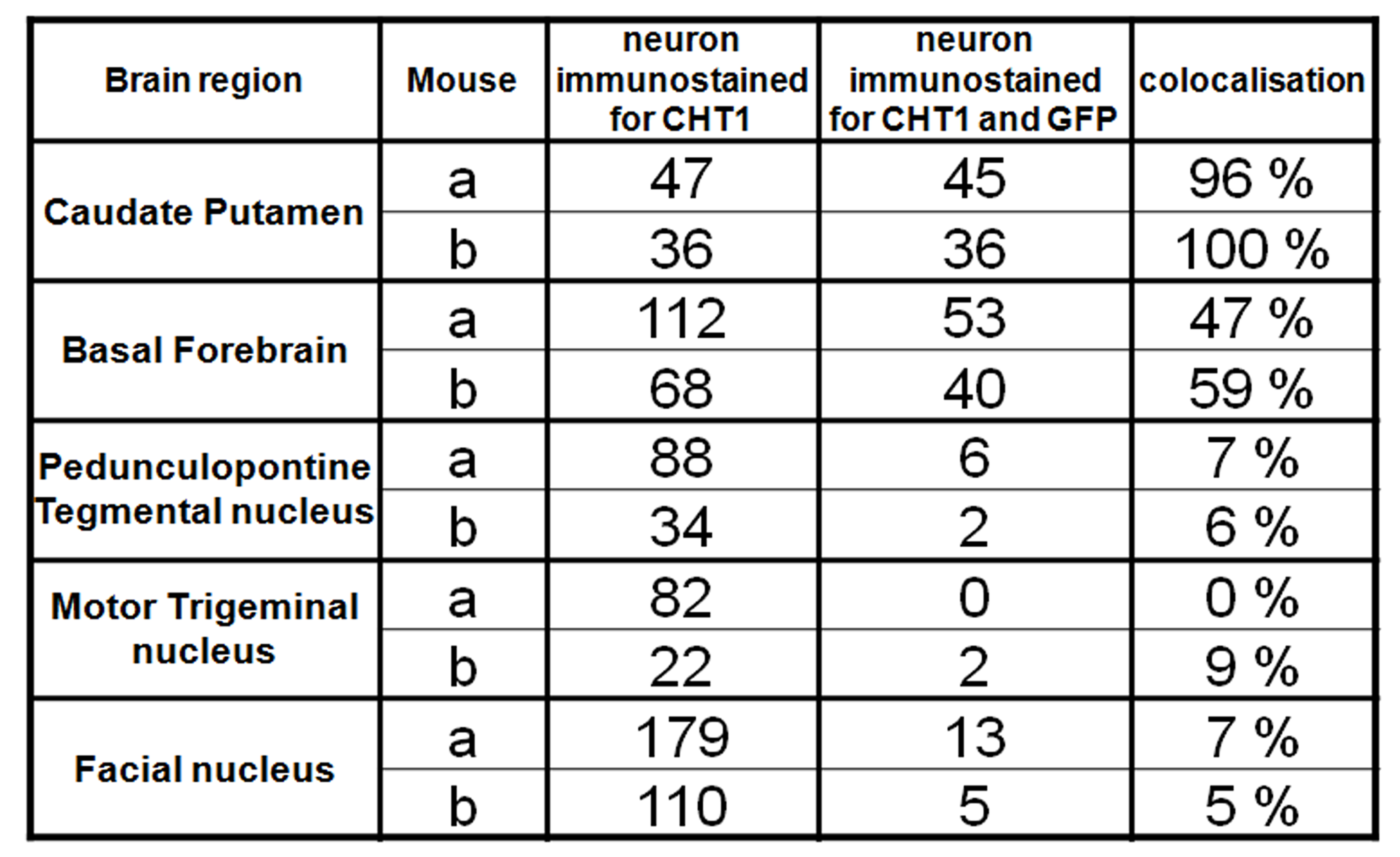

Supplement: Table S1 — Percentage of co-localization between cell with Cre-induced recombination and CHT1. (TIF) [file pbio.1001194.s007.tif]
